# Annotations

**Published:** 1902-03-15

**Authors:** 


### ANNOTATIONS.

### Hospital Rating.

The good service done by Mr. Burt and the Central Hospital Council was indicated in a measure by the proceedings of the London County Council in relation to local taxation at its last meeting. The Local Government and Taxation Committee recommended that the existing exemptions from rates should be abolished at the first opportunity. This point led to a considerable discussion, and after an earnest appeal from Dr. Collins in favour of the London hospitals, whose burdens he said were continuously increasing, an amendment was moved and carried that certain of these institutions should be exempted from rates. It is not easy to determine what effect this decision of the London County Council may have upon the rating of London hospitals at the moment, but it is fair to assume that the amendment will not be allowed to remain a dead letter, and that under Dr. Collins' able guidance the London County Council may use its influence to procure the early exemption of the principal London hospitals from rates. We have always been in favour of the managers of each hospital approaching the rating authority for their district with the object of inducing them to rate only those portions of the hospital building which are beneficially occupied. This course has been taken by a large number of the rating authorities all over the country, and results in rates being levied upon such hospital buildings only as are devoted to the staff. In any case, the thanks of the hospital managers are due to Dr. Collins, Mr. Crooks and Sir J. Dickson-Poynder for the strong case they made out for the exemption of the metropolitan hospitals from rating, which should prove fruitful in results in the near future.

### Tuberculous Children in Schools.

The education of consumptive children is a subject which is seriously engaging the attention of the Board of Education, and calls for further legislation. Hitherto some attempt has been made to deal with the problem of educating the unfit by the provision of special classes for epileptic and physically defective children who are incapable of deriving benefit from the ordinary instruction given in board schools; and the case of deaf and dumb children has also been specially cared for. But the presence of tuberculous children in schools opens up far different considerations. In the former cases it is the interest of the defective children themselves which has been regarded, and these special classes have been provided for them because of their inability to profit by the ordinary curriculum. But consumptive children are not necessarily "incapable of deriving benefit from such instruction," and therefore they do not come within the purview of the recent Act. Such is the opinion of a London magistrate who in a School Board prosecution the other day held that that Act afforded no excuse for the non-attendance at school of a consumptive child. He did, however, intimate that he thought it might be pleaded that such a child was not in a fit condition to mix with other children, and that its parents should not be penalised for keeping the child away. Having regard to the very decided opinion of the medical profession as to the infectious character of consumption, it is suggested that the time is ripe for some legislative provision to deal with this subject in the interest of the public health.

### Slums and Manslaughter.

When people hear of a murder being committed in a slum, they are apt to make excuses for the criminal on the ground that the conditions of life there are such as to encourage a tendency to crime. Overcrowding leads to enforced association with irritating neighbours; bad air induces a resort to drink, which makes an ordinarily peaceful man murderous in his inclinations, and so on. Even scientific criminologists sometimes declare that crime exists in direct proportion to the density of population. Yet, though it may be admitted that murders are more common in the lower and over-crowded parts of a town than in its better quarters, it does not follow that the mere fact of greater density of population should bear the whole blame. At least the statistics of crime seem to show that it is as dangerous to have too few neighbours as too many. Last year the Chicago *Times-Herald* collected the statistics of the year's murders in the United States. These showed that the smallest number of murders had been committed in Vermont—only six in the year—while at the other end of the scale stood Texas, with a record of 1,021. Texas is certainly larger than Vermont, which might explain part of the difference, but not all, if density of population is to be regarded as a factor, for Vermont is much the more thickly peopled of the two. Almost identical in the number of murders are Illinois, with 315; and Mississippi, with 317; but the latter has a population of only 1,650,000, as against the 5,800,000 persons who inhabit the former. Vermont's 350,000 inhabitants committed, as we have said, only six murders in the year, while among the 60,000 inhabitants of Nevada there were 29. The average number of murders in the five New England States is 248, while in California alone are found 422. These figures do not support the density of population theory; they oppose it. Of course one may say that in New England the bulk of the inhabitants spring from an orderly, God-fearing race, conscious of their everlasting responsibility for all their actions, and that they have almost no element in their midst like the negroes of the South, or the Spanish-Mexican half-breeds of California, to whom a bloodthirsty revenge for real or imagined wrongs seems natural; but is it not possible that in this connection the density of population theory cuts both ways? Is it not as dangerous for a man to have too few neighbours as too many; and may not a strong and wholesome public opinion, backed up by the presence of a policeman within easy call, help to teach the unenlightened the value of human life as much, as public parks and people's palaces? Not that these are to be despised, or any other means of making life happier and wholesomer for the poor; but we cannot yet afford to do without the policeman.

